# Transglutaminases in Monocytes and Macrophages

**DOI:** 10.3390/medsci6040115

**Published:** 2018-12-11

**Authors:** Huifang Sun, Mari T. Kaartinen

**Affiliations:** 1 Division of Biomedical Sciences, Faculty of Dentistry, McGill University, Montreal, QC H3A 0C7, Canada; huifang.sun@mail.mcgill.ca; 2 Division of Experimental Medicine, Department of Medicine, Faculty of Medicine, McGill University, Montreal, QC H3A 0C7, Canada

**Keywords:** transglutaminase, macrophage, monocyte, phagocytosis, osteoclast, microglia, atherosclerosis, inflammation

## Abstract

Macrophages are key players in various inflammatory disorders and pathological conditions via phagocytosis and orchestrating immune responses. They are highly heterogeneous in terms of their phenotypes and functions by adaptation to different organs and tissue environments. Upon damage or infection, monocytes are rapidly recruited to tissues and differentiate into macrophages. Transglutaminases (TGs) are a family of structurally and functionally related enzymes with Ca^2+^-dependent transamidation and deamidation activity. Numerous studies have shown that TGs, particularly TG2 and Factor XIII-A, are extensively involved in monocyte- and macrophage-mediated physiological and pathological processes. In the present review, we outline the current knowledge of the role of TGs in the adhesion and extravasation of monocytes, the expression of TGs during macrophage differentiation, and the regulation of TG2 expression by various pro- and anti-inflammatory mediators in macrophages. Furthermore, we summarize the role of TGs in macrophage phagocytosis and the understanding of the mechanisms involved. Finally, we review the roles of TGs in tissue-specific macrophages, including monocytes/macrophages in vasculature, alveolar and interstitial macrophages in lung, microglia and infiltrated monocytes/macrophages in central nervous system, and osteoclasts in bone. Based on the studies in this review, we conclude that monocyte- and macrophage-derived TGs are involved in inflammatory processes in these organs. However, more in vivo studies and clinical studies during different stages of these processes are required to determine the accurate roles of TGs, their substrates, and the mechanisms-of-action.

## 1. Introduction

Macrophages, which mean “big eaters” in Greek, are immune cells present in almost all tissues of the body with distinct tissue-specific phenotypes and functions. The highly heterogeneous macrophages have multiple origins: yolk sac, fetal liver, and circulating monocytes [1]. Macrophages play a central role in tissue homeostasis as well as in inflammation via phagocytosis and orchestrating immune responses [2]. Macrophages kill the ingested pathogens via phagocytosis and protect the organism from infection. Macrophages can also remove apoptotic cells by efferocytosis, a specialized form of phagocytosis, to prevent the induction of inflammation by the apoptotic cells [3]. In addition to their role as guardians, macrophages play an essential role in organizing inflammatory reactions by producing pro- and anti-inflammatory mediators and effector molecules such as chemokines, cytokines, and growth factors [2]. Consistent with such a broad array of functions, macrophages are involved in various inflammatory disorders and pathological conditions, such as atherosclerosis, osteoporosis, neurodegenerative disorders, and autoimmune diseases [4].

The transglutaminase (protein glutamine-γ-glutamyltransferase, TGase, TG, EC 2.3.2.13) family consists of nine structurally and functionally related proteins. Of the nine TGs, one (erythrocyte membrane protein band 4.2) lost its catalytic activity in evolution and acts as a structural protein, and eight (TG1-7 and Factor XIII-A (FXIII-A)) are active enzymes showing the Ca^2+^-dependent transamidation and deamidation activity [5]. In addition to this enzymatic activity, some TGs may function as atypical GTPase and ATPase, protein disulfide isomerase, protein kinase, and also have other non-enzymatic functions in cell signaling and cell-matrix interactions [6,7,8]. Numerous studies have shown that TGs, particularly TG2 and FXIII-A, are extensively involved in inflammatory processes, making them useful markers for disease diagnosis or potential therapeutic targets [9,10,11]. In this review, we outline the existing data on expression and regulation of TGs in macrophages and elaborate on the distinct functions of TGs in monocytes and tissue-specific macrophages to improve our understanding of their contributions to various diseases.

## 2. Brief Overview of Transglutaminase Family Members

Transglutaminase 2, also named as tissue TG due to its ubiquitous expression in cells and tissues, is the most studied member of the family. Mainly a cytosolic protein, it is also found in the nucleus, on the plasma membrane, as well as in the extracellular matrix [12]. Studies show that P2X7 receptor, nitric oxide, and perinuclear recycling endosomes regulate TG2 externalization [13,14,15,16] and that TG2 binding to cell surface heparan sulfate proteoglycans is critical for its translocation to the cell surface [17]. Being an extremely versatile protein, TG2 exhibits transamidation, GTPase, ATPase, protein kinase, and protein disulfide isomerase activity. It is involved in various disease processes such as celiac disease and neurodegenerative disorders [8].

As the last zymogen activated in the blood coagulation cascade, FXIII-A catalyzes the stabilization of newly formed fibrin network. Thus, FXIII-A is crucial to maintain hemostasis and its deficiency results in severe bleeding diathesis. It is present in platelets, monocytes, macrophages, dendritic cells, chondrocytes, osteoblasts, osteocytes, and adipocytes [18,19]. Recent study shows resident macrophages are the major source of plasma FXIII-A [20]. In addition to its role in hemostasis, FXIII-A also has a pivotal role in maintenance of pregnancy, angiogenesis, wound healing, and bone and energy metabolism [21,22].

Transglutaminase 1, also known as keratinocyte TG, is primarily expressed in the stratified squamous epithelia of the integument, the upper digestive tract, and the lower female genital tract. It is required for the formation of cornified cell envelope that acts as protective skin barrier [8]. Mutations in *TGM1* cause lamellar ichthyosis in humans, an autosomal recessive skin disorder [23,24,25].

Transglutaminase 3, also referred to as epidermal TG, is found in hair follicles, epidermis, and brain. Analogous to TG2, TG3 can also bind to and hydrolyze GTP [8]. It is required for hair fiber morphogenesis [26].

Transglutaminase 4, also known as prostate TG, is a prostate-specific transglutaminase and present in the seminal plasma [8]. It is required to form the copulatory plug in mice and the defects in copulatory plug formation lead to reduced fertility [27]. TG4 is also a prospective marker of prostate cancer progression [28].

Transglutaminase 5 is widely expressed in the epidermis and the loss-of-function mutations of *TGM5* result in skin peeling syndrome in humans [29]. It is also involved in the hyperkeratosis in ichthyosis and psoriasis patients [30]. The crosslinking activity of TG5 can be inhibited by GTP and ATP [8].

Transglutaminase 6 and TG7 have a similar expression pattern which is restricted to testes, lungs, and brain [8]. Mutations in *TGM6* cause spinocerebellar ataxia type 35, a rare autosomal dominant neurodegenerative disease [31]. The function of TG7 remains unclear.

Some studies suggest that TGs may be able to compensate for each other’s functions partially in tissues where their expressions and activities overlap [32,33,34,35].

## 3. Development and Classification of Macrophages

Blood monocytes were historically considered as a reservoir for macrophages [36,37], however, in recent years, this paradigm has shifted dramatically [38]. At steady state (i.e., without inflammatory cues), the contribution of monocytes to macrophages is only restricted to a few specific tissues including the intestine, dermis, heart, and pancreas [39]. Numerous studies have determined that some macrophages and their precursors are embryonically established in the yolk sac and fetal liver before the generation of the first hematopoietic stem cells (HSCs) [40,41,42,43,44,45]. Tissue macrophages can maintain themselves through self-renewal during adulthood with minimal monocyte input [46,47,48,49,50]. Development, proliferation, differentiation, and function of macrophages are regulated by two growth factors: colony stimulating factor 1 (CSF-1), also known as macrophage-colony stimulating factor (M-CSF), and interleukin-34 (IL-34) [51].

Macrophages are distinguished as large vacuolar cells and they are phenotypically defined positive for F4/80 in mice [52]. Macrophages can be classified based on their tissue of residence and activation. Governed by tissue specific cues, macrophages can adapt to different milieus, such as splenic macrophages, peritoneal macrophages, alveolar and interstitial macrophages (lung), Kupffer cells (liver), osteoclasts (bone), and microglia (central nervous system, CNS) [51]. Macrophages can also respond to different activation signals (cytokines or bacterial products) with changes in their morphology, phenotype, transcriptome, and function. Macrophage activation occurs in two modes, i.e., classically activated (M1) and alternatively activated (M2) macrophages. M1 is activated by lipopolysaccharide (LPS)/interferon gamma (IFN-γ) and exhibits pro-inflammatory features, whereas M2 results from IL-4/IL-13 stimulation and displays anti-inflammatory properties [53]. M1 and M2 perform diametrically opposed functions by generating either nitric oxide (NO) or ornithine via different cleavages of terminal nitrogen linkages of arginine. M1 performs killing/inhibitory functions by producing NO, which inhibits cell proliferation and kills microbes, while M2 displays healing/repair capacities via generating ornithine, which promotes cell proliferation and repair [54].

## 4. Transglutaminases in Monocyte Adhesion and Extravasation

As mentioned above, the majority of macrophages are seeded before birth and exhibit negligible need for monocytes throughout adulthood due to their ability of self-proliferation [39]. However, monocyte recruitment into tissues and monocyte-derived macrophages are strongly increased during inflammation [55]. In order to reach the inflamed sites and exploit their functions, cell adhesion and extravasation are required for circulating monocytes. Over 30 years ago, TG2 and FXIII-A were found present in monocytes [56,57]. Akimov et al. demonstrated that both TG2 knock-down and inhibition of TG2-fibronectin binding by function-blocking antibodies decreased the adhesion and spreading of monocytes on fibronectin [58]. In addition, antibodies against β1 and β3 integrins resulted in the similar inhibition but in a less prominent manner. Notably, antibody against FXIII-A had no such effects, suggesting that only TG2 is involved in adhesion and spreading of monocytes [58]. In addition, studies showed TG2 expression was highly upregulated in monocytes during their adhesion onto endothelial cells, indicating that TG2 is required for monocyte extravasation [59]. However, in another study FXIII-A was shown to have a role in monocytes, where it catalyzed the dimerization of angiotensin II type 1 receptor (AT_1_ receptor) on monocytes. This FXIII-A-dependent dimeric form of the AT_1_ receptor displayed enhanced signaling and increased monocyte adhesion to endothelial cells in hypertensive patients [60]. Therefore, both TG2 and FXIII-A contribute to the monocyte adhesion and extravasation but via different mechanisms.

## 5. Transglutaminases in Macrophages

### 5.1. Expression and Regulation of Transglutaminases in Macrophages

Over 30 years ago, TG2 and FXIII-A were also found present in macrophages [61,62,63,64] and both of them were identified as novel markers for alternatively activated M2 macrophages [65,66]. Numerous studies demonstrated that level of TG2 increased dramatically during the differentiation from monocytes to macrophages, induced either by adherence to the cell culture dish or by several macrophage stimulating factors such as serum retinoids, IFN-γ, LPS, and 12-O-tetradecanoyl-phorbol-13-acetate (TPA) [56,67,68,69]. In addition, various stimulating factors participate in the regulation of TG2 expression in differentiated macrophages. Numerous studies demonstrated that retinoic acid was able to induce TG2 expression in macrophages [70,71,72,73] and this induction involved cyclic AMP and protein kinase C-dependent pathway [74,75]. Ghanta et al. showed that LPS was a potent inducer of TG2 expression in macrophages and this induction was co-regulated by metastatic tumor antigen 1 (MTA1) and nuclear factor kappa-light-chain-enhancer of activated B cells (NF-κB) signaling [76]. In response to LPS stimulation, there was an enhanced recruitment of NF-κB subunit p65-MTA1-pol II complex onto the −630 to −849 region of TG2 promoter [76]. Falasca et al. showed that LPS induced an enhanced NF-κB activation in macrophages in a TG2-dependent manner, therefore, upon LPS treatment, the induction of TG2 in macrophages resulted in a loop of continuous activation of NF-κB pathway. However, TG2 null macrophages treated by LPS lost this activity to activate NF-κB [77]. In addition to activate NF-κB, the LPS induced TG2 in macrophages also mediated an elevated activity of phospholipase A2 (PLA2), which catalyzes the production of arachidonic acid (AA), an important substance during inflammation [78]. Sarang et al. showed that TG2 null macrophages responded to LPS treatment with increased production of IL-6 and tumor necrosis factor alpha (TNFα) due to an elevated integrin α_v_β_3_-mediated Src kinase activity [79]. However, Yoo et al. demonstrated that TG2 null mice treated with LPS displayed reduced levels of IFN-γ and IL-6 in the serum [80]. This contradiction may arise from the discrepancy between in vivo and in vitro studies. Intriguingly, in addition to the upregulation of TG2 by pro-inflammatory LPS, anti-inflammatory mediator IL-4, also the M2 macrophage inducer, has been shown to upregulate TG2 expression in macrophages as well [81,82,83]. However, the IL-4 induced expression of TG2 was blunted in estrogen receptor α (ERα) deficient macrophages due to the presence of a full consensus estrogen response element (ERE) in the TG2 promoter [83]. Moreover, IL-6 is also an inducer of TG2 expression in macrophages [84]. Additionally, transforming growth factor β1 (TGF-β1) induced TG2 expression in macrophages, and TG2 was also found to promote TGF-β1 expression [85,86]. Notably, there were several studies showing contradictory results on the TG expression during macrophage differentiation. Two studies revealed that TG2 increased and FXIII-A decreased during macrophage differentiation [87,88]. Consistently, there was an upregulation of cell surface TG2 associated with β1 and β3 integrins, while no association of FXIII-A with integrins was detected and a simultaneous decrease of surface FXIII-A during macrophage differentiation was seen [58]. However, on the contrary, another three studies confirmed the increase of FXIII-A during macrophage differentiation [65,89,90]. This contradiction may arise from the use of different macrophage models in vitro. In addition, Adany et al. showed that there was a transient intranuclear accumulation of FXIII-A in the early phase of macrophage differentiation and FXIII-A in the nuclei also exhibited TG activity [91]. Studies also showed that FXIII-A was strongly induced in macrophages by the combination of IL-4 and dexamethasone, but not by either of them alone [81]. Our work demonstrated, for the first time, that TG1 was also expressed in macrophages and M-CSF strongly upregulated its expression [92,93].

Taken together, TG2, FXIII-A, and TG1 are all present in macrophages and their expressions are upregulated during macrophage differentiation. TG2 can be induced by a plethora of factors, such as pro-inflammatory LPS and anti-inflammatory IL-4, suggesting TG2 is involved in both induction and resolution stage of inflammation. It can also be induced by factors with both anti- and pro-inflammatory properties such as IL-6 and TGF-β1 [94,95], indicating that TG2 may exert distinct functions in response to the same factor under various circumstances.

### 5.2. Transglutaminases in Macrophage Phagocytosis

Phagocytosis is restricted to specialized cells, including professional phagocytes (neutrophils, monocytes, macrophages, and dendritic cells) or non-professional phagocytes (fibroblasts, endothelial, and epithelial cells). It is a receptor-mediated and actin-polymerization-dependent process for internalizing particles greater than 0.5 µm in diameter [96]. The target particles include the apoptotic cells, necrotic cell debris, and opsonized pathogens [97]. The engulfment of apoptotic cells by macrophages, also termed as efferocytosis, is crucial for tissue homeostasis, resolution of inflammation, and embryologic development [98].

The involvement of TG2 in macrophage phagocytosis was initially reported in 1981 [99], and was further confirmed by various in vitro studies [100,101]. Subsequently, studies showed that TG2 null mice displayed defective clearance of apoptotic thymocytes by macrophages in the thymus [102] and impaired clearance of apoptotic hepatocytes by Kupffer cells in the liver [102,103]. However, TG2 null macrophages exhibited unimpaired ability to ingest bacteria, yeast, opsonized non-apoptotic thymocytes [102], monosodium urate crystals [104], or oxidized low-density lipoproteins [105]. Additionally, TG2 null mice showed inflammatory infiltrates at the apoptosis sites in short term [102,103] and then developed autoimmunity in the long term [102]. Two other studies confirmed that TG2 was required for the engulfment of apoptotic cells by macrophages, but not for their recognition and binding [103,104]. These studies also confirmed that TGF-β, a cytokine which is released by macrophages engulfing apoptotic cells [106] and whose activation requires TG2 [107], was involved in the defective phagocytosis in TG2 null macrophages [102,103,104] and exogenous active TGF-β1 was able to rescue this phagocytosis defect [104]. Subsequent studies revealed that extracellular or cell surface localization of TG2 was responsible for the TG2-mediating phagocytosis and that its crosslinking function was not required for this since exogenous recombinant TG2, both wild type and catalytically inactive forms, was able to rescue the defective phagocytosis in TG2 null macrophages. Moreover, the function of TG2 in phagocytosis is dependent on its guanine nucleotide-binding pocket [104,108]. In addition, Toth et al. showed that TG2 formed a complex with milk fat globulin EGF factor 8 (MFG-E8) and integrin β3 on macrophage surface and was required for the formation of engulfing portals. In addition, there was a compensatory increase in integrin β_3_ expression in the TG2 null macrophages that partially corrected the impaired integrin β3 signaling caused by the absence of TG2 [108,109]. In contrast to the previous findings, studies showed that TG2 crosslinking activity on the cell surface was critical for macrophage recruitment to, binding and removal of apoptotic cells, and suggested that TG2, in association with syndecan-4 on cell surface, promoted clearance of apoptotic cells by crosslinking CD44 [110]. More recently, studies unveiled a role of TG2 in regulating levels of phagocytosis-related molecules CD14 and class A macrophage scavenger receptor type I (SR-AI) in macrophages [88]. However, upregulation of TG2 alone in macrophages is not sufficient to promote their capacity to remove apoptotic cells [111]. Intriguingly, studies showed that both receptors for the Fc region of IgG (FcγR)- and complement receptor (CR)-mediated phagocytosis were strongly diminished in monocytes of FXIII-A deficient patients [89]. In addition, a human myelomonocytic cell line, negative for FXIII-A and incapable of phagocytosis, displayed FcγR- and CR-mediated phagocytosis and FXIII-A expression after differentiation to macrophages induced by TPA treatment [112].

To conclude, TG2 on the cell surface or in the extracellular matrix plays an essential role in macrophage phagocytosis of apoptotic cells via interaction with integrin β3 or other molecules/receptors. The defective clearance of apoptotic cells resulting from the absence of TG2 is associated with inflammation and autoimmune diseases. FXIII-A is involved in the FcγR- and CR-mediated phagocytosis in macrophages.

### 5.3. Transglutaminases in Vascular Macrophages

Atherosclerosis is an immunoinflammatory vascular disorder characterized by the build-up of lipids, cholesterol, calcium, and cellular debris on the intima of medium-sized and large arteries [113]. It is well established that monocytes and macrophages play important roles in the initiation and progression of atherosclerosis [114], and that both TG2 [115,116] and FXIII-A [117] are present in human atherosclerotic plaques. Additionally, FXIII-A was found to be the dominant TG in human atherosclerotic arteries, and it may be derived from the local macrophages [118]. Studies demonstrated that irradiated low-density lipoprotein receptor (LDLR) knockout mice followed by TG2−/− bone marrow transplantation (BMT) displayed larger and deeper atherosclerotic aortic valve lesions compared with TG2+/+ BMT mice, indicating that leukocyte-derived TG2 limited the progression of atherosclerosis [105]. In this study, authors suggested that macrophage-derived TG2 may have exerted its functions by (a) promoting the phagocytosis of apoptotic cells, (b) modulating production of TGF-β which is involved in plaque stability, and (c) regulating the expression of ATP-binding cassette transporter A1 (ABCA1) which mediates reverse cholesterol transport [105]. Another study showed that TG2 deficient mice exhibited a decreased collagen content and increased macrophage content in the atherosclerotic plaques, indicating a more unstable and more rupture-prone plaque [119]. In this study, FXIII-A was expressed in atherosclerotic plaques in TG2 deficient mice and suggested to compensate for the loss of TG2. FXIII-A is likely derived from macrophages and is sufficient to prevent plaque rupture by crosslinking the matrix proteins [119]. In contrast to the anti-atherogenic role of macrophage-derived TG2 and FXIII-A in later stages of atherosclerosis, studies showed that TG2 null mice displayed significantly less monocytes/macrophages on the carotid artery when exposed to oscillatory shear stress [120]. Additionally, FXIII-A inhibition by expression of the FXIII-A inhibitor FXIII-A^N73-D98^ decreased monocyte/macrophage infiltration of the aorta, leading to inhibition of atherosclerosis development in hypercholesterolemic apolipoprotein E deficient mice [60]. Furthermore, systemic inhibition of both TG2 and FXIII-A with the TG inhibitor L682777 did not alter the lesion size, but resulted in reduced macrophage content in the media of vessels, suggesting that TG2 and FXIII-A contribute to the early development of plaque formation via directly affecting monocyte/macrophage infiltration [121]. In conclusion, macrophage-derived TG2 and FXIII-A play an anti-atherogenic role in later stages of atherosclerosis, whereas TG2 and FXIII-A show pro-atherogenic function in the early stages of atherosclerosis by regulating monocyte/macrophage infiltration.

### 5.4. Transglutaminases in Alveolar and Interstitial Macrophages

In the lung, there are two major populations of macrophages present, alveolar and interstitial macrophages. Alveolar macrophages reside within the lumen of alveolus, whereas interstitial macrophages locate to the interalveolar spaces [122]. Studies showed that cigarette smoke inactivated TG2 by attacking the active-site cysteine residue via an oxidative mechanism in alveolar macrophages [123,124]. In addition, there was a decrease in TG2 activity in the alveolar macrophages of smokers compared to nonsmokers [125]. Another study showed that TG2 inhibition decreased the production of cysteinyl leukotriene (a product derived from AA) in IL-4-treated macrophages, suggesting a role for TG2 in airway inflammation [126]. FXIII-A is also present in alveolar macrophages [127]. The levels of FXIII-A derived from activated or injured alveolar macrophages were elevated in the bronchoalveolar lavage fluid from patients with chronic bronchoalveolar inflammation [128]. The role of TGs in interstitial macrophages is an interesting topic but there is a paucity of information. The limited available literature showed that lung interstitial macrophages from the ovalbumin-induced asthmatic mice were polarized towards the M2 phenotype with an increased expression of TG2, which is a marker of M2 macrophages [129]. These findings suggest that macrophage-derived TG2 and FXIII-A play a role in airway inflammation, but further research is required to determine their accurate functions.

### 5.5. Transglutaminases in Microglia

Microglia, resident macrophages in the central nervous system (CNS), represent 5%–20% of the total glial cell population [130]. Microglia have multiple functions and are involved in various pathological processes, such as neuroinflammation, neurodegeneration, ischemia, and trauma [131]. Microglia are usually maintained in a resting or relatively inactive state with a “ramified” morphology with many short and fine processes. These processes undergo continuous cycles of extension and retraction to scan their local environment [132]. In response to trauma, infection, or infarction, microglia are activated and transform into “amoeboid” morphology (i.e., spherical shape without processes) and display phagocytosis [132]. These cells are morphologically indistinguishable from infiltrated monocytes/macrophages in the CNS.

Alzheimer’s disease (AD) is characterized by neuronal loss and accumulation of pathogenic amyloid-β (Aβ) assemblies in the brain, and this Aβ-induced neuronal loss was caused by the enhanced microglial phagocytosis [133]. In addition, increased NO production of microglia also resulted in neuronal loss in neurodegenerative diseases [134]. Studies showed that LPS increased TG2 expression in BV-2 microglia. This induction of TG2 promoted the NO production and enhanced the phagocytosis of dead cells in microglia [135,136] via the TG2-mediated activation of NF-κB pathway [137]. In addition, amphotericin B upregulated the expression of TG2 and inducible NO synthase (iNOS) and increased the phagocytosis of dead cells in BV-2 microglia [138]. Recent studies showed that the uptake of Aβ by microglia was mediated by forming a complex of aggregated Aβ/MFG-E8/TG2 [139]. In order to explore the roles of TG2 in the pathogenesis of AD, many studies were carried out using THP-1 cells, a monocyte-like cell line. Studies showed that Aβ treatment increased TG2 expression in THP-1 cells and TG2 was required for the Aβ-induced monocyte maturation and activation [140]. Another study confirmed that TG2 mediated the Aβ-induced THP-1 monocyte activation via AP1/JNK signaling pathways [141]. Additionally, FXIII-A positive microglia were found abundant in primitive plaques in parietal cortex of AD brains, suggesting FXIII-A plays a role in the early stage of AD pathology [142].

Multiple sclerosis (MS) is an inflammatory demyelinating CNS disease, characterized by sensory, motor, and cognitive deficits [143,144]. The principal study model for MS is the experimental autoimmune encephalomyelitis (EAE) [145]. In marmosets with EAE, TG2 was expressed by infiltrating monocytes in active white matter lesions. In addition, TG2 was co-localized with integrin β_1_ and closely associated with extracellular fibronectin, suggesting that TG2 plays a crucial role in the adhesion and migration of infiltrating monocytes in EAE. In contrast to the white matter lesions, TG2 was expressed mainly by resident microglia in grey matter lesions. However, fibronectin expression was absent suggesting an alternative role for microglia-derived TG2 in grey matter lesions [146]. Studies also confirmed the presence of TG2 in infiltrated macrophages in human MS lesions [147]. Furthermore, in rats with EAE, inhibition of TG2 activity resulted in clinical improvement and attenuated demyelination, indicating a role for TG2 during MS pathogenesis [147]. To determine if the crosslinking activity of TG2 caused these effects, TG2 inhibitors, BJJF078 and ERW1041E (neither of them interferes with TG2 binding to fibronectin), were administered in this model. Studies showed that only BJJF078 inhibited cellular TG2 activity in THP-1 cells and only ERW1041E resulted in attenuated EAE disease motor-symptoms, suggesting that extracellular TG2 activity, rather than cellular activity, is more likely involved in mouse EAE pathology [148]. In a newly developed mouse model which enables the in vivo visualization of monocytes during EAE, TG2 was present in monocytes in the spinal cord lesions and TG2 positive monocytes attached to the endothelial lumen of the blood vessel, suggesting that TG2 may contribute to the extravasation of monocytes into the CNS during EAE [149].

Intriguingly, recent studies revealed that microglia-derived TG2 promoted myelin formation and repair by oligodendrocytes [150]. Microglia-derived TG2 was also associated with neuronal death induced by ischemia/reperfusion [151]. In addition, studies showed that oligomerization of superoxide dismutase 1 catalyzed by TG2 induced activation of BV-2 microglia, accelerating neuroinflammation in amyotrophic lateral sclerosis (ALS) [152]. Furthermore, studies showed hyperhomocysteinemia was associated with various chronic neurodegenerative diseases, such as AD, MS, and ALS, and TG2 played an essential role in homocysteine-induced activation of THP-1 monocytes [153].

Collectively, these findings reveal a contributing role of TG2 to the pathogenesis of various neurological disorders such as AD, MS, and ALS via regulating phagocytosis and activation of microglia and affecting monocyte infiltration into the CNS.

### 5.6. Transglutaminases in Osteoclasts

Bone is a mineralized form of connective tissue and its integrity is maintained by constant remodeling through the balanced activities of two cell types: the bone-forming osteoblast and the bone-degrading osteoclast, a specialized bone-resident multinucleated macrophage [154]. Starting from monocytes/macrophages, osteoclast differentiation requires two essential cytokines, M-CSF and receptor activator of nuclear factor-kappaB ligand (RANKL). Osteoclastogenesis is a complex process involving a differentiation stage, followed by a cell fusion and multinucleation stage [155]. When osteoclasts attach to bone, they generate two polarized structures: the bone-apposed ruffled border and the sealing zone which seals off a resorption compartment between the cell membrane and the bone surface. Protons are pumped and proteolytic enzymes are secreted from the ruffled border side of the osteoclast into the resorption compartment to dissolve minerals and degrade bone matrix proteins [156]. Increased osteoclast activity is responsible for bone destruction in diseases such as osteoporosis, periodontitis, and rheumatoid arthritis [157]. Recently, four studies were published by us and others exploring the role of TGs in osteoclastogenesis and bone resorption. Raghu et al. showed that FXIII-A deficient mice displayed reduced osteoclastogenesis in vivo and in vitro and that TG inhibitor, cystamine, suppressed osteoclastogenesis in vivo [158]. Furthermore, our work showed that TG2 and FXIII-A double knockout mice exhibited severe osteopenia due to increased bone resorption. In addition, the double FXIII-A/TG2 deficient macrophages gave rise to increased osteoclastogenesis in vitro, suggesting that the two enzymes negatively regulate osteoclastogenesis [92]. However, a non-specific TG inhibitor, NC9, blocked osteoclastogenesis in this study, leading to the discovery of TG1 being expressed in wild type and FXIII-A/TG2 deficient macrophages and osteoclasts [92]. Subsequently, Kim et al. showed that suppressed expression of TG2 by siRNA resulted in increased osteoclastogenesis in vitro and that TG2 deficient mice displayed increased osteoclast number and lower trabecular bone mass in vivo [159]. However, we did not see this phenotype in TG2 knockout mice [92]. Most recently, our work showed that NC9 inhibited differentiation, migration, and fusion of pre-osteoclasts, as well as resorption of mature osteoclasts. NC9 also increased RhoA levels and blocked podosome belt formation, suggesting TG activity regulates actin dynamics in osteoclasts. Consistently, this study showed that TG1, TG2 and FXIII-A co-localized to podosomes in osteoclasts [93]. Furthermore, studies showed that Z006 (a TG inhibitor) became cell permeable at 40 µM [160], and Z006 only exhibited inhibitory effect on osteoclast differentiation at 40 µM or higher concentrations, indicating that intracellular TG activity is involved in osteoclastogenesis [93]. Another two interesting studies revealed an indirect role of TG2 in regulating osteoclast differentiation. One study showed that TG2 inhibition resulted in reduced RANKL expression in macrophages and human periodontal ligament cells [161]. Another study showed that TG2 mediated 1α,25-dihydroxyvitamin D_3_-induced macrophage fusion in a spermidine-dependent mechanism [162]. Although these findings may seem contradictory, they are not incompatible. Since three TGs are present in osteoclasts whose differentiation involves several cellular events, it is possible that they all have separate functions, and/or may be up- or downregulated in the absence of others, which would complicate interpretation of null mouse models. Future work should be directed to dissect out their separate functions and joint effects on osteoclasts.

### 5.7. Transglutaminases in Other Macrophages

Studies showed that pro-inflammatory LPS and IFN-γ induced secretion of thioredoxin-1 (TRX) by macrophages was able to activate the extracellular TG2 in macrophages in vitro and that intravenous administration of TRX resulted in a rapid increase in TG2 activity exclusively in the small intestine in mice (i.e., this TRX-induced TG2 activity was not observed in other organs), suggesting that enhanced TG2 activity may be caused by inflammation in celiac disease [163]. Previous studies showed that crosslinking activity of TG2 was required for apoptotic envelope formation on macrophages infected with attenuated *Mycobacterium tuberculosis* (MTB) strain and thus the apoptotic macrophages limited bacterial replication and dissemination [164]. Recent studies showed that both inactivation and inhibition of TG2 suppressed the intracellular replication of virulent MTB in macrophages via impairing cell autophagy [165]. Another two interesting studies showed that cysteamine, a TG inhibitor (albeit not specific) [166], improved the autophagy of macrophages derived from patients or mice with cystic fibrosis, resulting in enhanced bacterial clearance by macrophages [167,168]. Studies also demonstrated that peritoneal injection of cystamine reduced the activity of matrix metalloproteinase-9, the expression of TNF-α and TGF-β in macrophages, and suppressed the production of anti-cardiolipin autoantibody in mice with systemic lupus erythematosus [169].

## 6. Conclusions

Macrophages are highly heterogeneous immune cells found in multiple tissues. Monocytes, previously considered as the immediate upstream precursors of macrophages, infiltrate into tissues upon damage or infection. Depending on the tissue context, they exert distinct functions, which can considerably overlap with tissue-resident macrophages. Over the last 30 years, numerous studies suggest that TGs, particularly TG2 and FXIII-A, participate in monocyte- and macrophage-mediated physiological and pathological processes. Firstly, TG2 and FXIII-A contribute to monocyte adhesion and extravasation, a process essential for the initiation of inflammation, especially for the pathogenesis of atherosclerosis and MS. Next, TG2, FXIII-A, and TG1 are all present in macrophages and their expressions are upregulated during macrophage differentiation. TG2 can be induced by both pro-inflammatory and anti-inflammatory substances, suggesting TG2 is involved in both induction and resolution stage of inflammation. Among these mediators, LPS is the most important one due to its association with septic shock [77,80]. Furthermore, TG2 and FXIII-A play critical roles in different types of macrophage phagocytosis. TG2 on the macrophage surface or in the extracellular matrix interacts with integrin β3 or other molecules/receptors to mediate phagocytosis of apoptotic cells, a process crucial for resolution of inflammation and for prevention of development of autoimmune diseases. FXIII-A is involved in FcγR- and CR-mediated phagocytosis in macrophages. Finally, we reviewed the roles of TGs in tissue-resident macrophages and infiltrated monocytes in different tissue environments. In vasculature, macrophage-derived TG2 and FXIII-A play an anti-atherogenic role in later stages of atherosclerosis, whereas TG2 and FXIII-A show pro-atherogenic function in the early stages of atherosclerosis by regulating monocyte/macrophage infiltration. In the lung, macrophage-derived TG2 and FXIII-A play a role in airway inflammation, but further research is required to accurately determine their functions. In CNS, TG2 participates in the pathogenesis of various neurological disorders such as AD, MS, and ALS via regulating phagocytosis and activation of microglia and affecting monocyte infiltration. In bone, TG2, FXIII-A, and TG1 are all present in osteoclasts. Although different mouse models show contradictory results regarding the role of TGs on osteoclast differentiation, in vitro studies show TG activity is required for osteoclastogenesis. Future work should be directed to dissect out their separate functions and joint effects on osteoclasts. However, the mechanisms of action of TGs in these processes are not yet fully understood. Thus, further research is needed to determine the extracellular or intracellular localization of TGs, the catalytic or non-catalytic activities involved, and target substrates in monocytes and macrophages. This will benefit from many advancements in currently available TG research tools, including TG-specific inhibitors [160,170,171,172,173,174] and substrate peptides [175,176,177,178,179,180].
